# Biological Properties and Bioactive Components of *Mentha spicata* L. Essential Oil: Focus on Potential Benefits in the Treatment of Obesity, Alzheimer's Disease, Dermatophytosis, and Drug-Resistant Infections

**DOI:** 10.1155/2019/3834265

**Published:** 2019-10-20

**Authors:** Mohammed S. Ali-Shtayeh, Rana M. Jamous, Salam Y. Abu-Zaitoun, Ahmad I. Khasati, Samer R. Kalbouneh

**Affiliations:** Biodiversity and Environmental Research Center (BERC), Til, Nablus, State of Palestine

## Abstract

In the present study, the medicinal aromatic plant *Mentha spicata* has been investigated as a source of essential oil (EO) and pharmaceuticals. The quantity and composition of EO from *M. spicata* cultivated in Palestine were analyzed seasonally over a three-year period. A significantly higher EO content was produced in summer and fall months (2.54–2.79%). Chemical analysis of EO revealed 31 compounds with oxygenated monoterpenes (90%) as the most abundant components followed by sesquiterpene and monoterpene hydrocarbons (6 and 3%, respectively). *M spicata* can be characterized as a carvone chemotype (65%). EO and carvone have shown strong inhibitory activities against the principal enzymes associated with Alzheimer's disease (AD) and overweight diseases (cholinesterase and porcine pancreatic lipase) and also shown strong antidermatophytic activity against *Microsporum canis*, *Trichophyton rubrum*, *T. mentagrophytes*, and *Epidermophyton floccosum*. The pancreatic lipase inhibition and the synergism showed the potential activity of *M. spicata* EO and carvone and that their combinations with standard drugs can be useful for the treatment of obesity and overweight. The results also demonstrated that, in addition to their significant inhibitory activity against biofilm formation of methicillin-resistant *Staphylococcus aureus* (MRSA), *M. spicata* EO and carvone had a strong inhibitory effect on metabolic activity and biomass of the preformed biofilm. The current study supports the utilization of *M. spicata* EO as a traditional medicine and opens perceptions to find more potent substances in the EO for the management of obesity, AD, and dermatophytosis and for combating drug-resistant bacterial infections.

## 1. Introduction

Spearmint (*Mentha spicata* L.) is a cultivated perennial, rhizomatous, and glabrous plant of the Lamiaceae family and is one of the most common herbs grown commercially worldwide including Palestine. It has been considered one of the most important essential oil crops [1]. Spearmint fresh and dried leaves are used as teas and spice and to flavor foods, dishes, and beverages. Herbage, extracts, and essential oil (EO) of the plant have long been used for combating a number of human diseases or relieving ailments [2].

Spearmint EO is used widely as an aromatic agent in numerous products such as chewing gum, dental cream, and mouth washes, as well as in medications, sweet, fragrance, and ecological pesticides and as antimicrobial agents [3]. The plant is considered a source material for EO that has been found to be a valuable source of natural phenolic antioxidants, cholinesterase inhibitors, pancreatic lipase inhibitors, and biofilm disinfection, antifungal, and antiproliferative agents [4–8].

The most dominant constituent found in spearmint oil worldwide is R-(*−*)-carvone, which offers spearmint its unique smooth characteristic scent [9]. *M. spicata* oil also contains noteworthy concentrations of limonene, dihydrocarvone, and 1,8-cineole [10–12]. However, carvone is reported to be potential in bacterial growth inhibition, as well as reported as a fungicide, insect repellent, and potato- or flower bulb-sprouting suppressant [13].

In recent years, due to the increasing interest in natural products, plant EOs have attracted more attention in phytomedicine because of their extensive diversity of bioactivities [14–17]. Furthermore, researches have revealed synergistic effects of standard drugs when applied in combination with specific EOs or some of their main constituents, responsible for exerting such activities [18–25]. Such investigations indicate that the combination of EOs with standard drugs provides significant potential for enhancing the therapeutic effect of existing therapies, developing novel strategies for the management of infectious diseases caused by multidrug-resistant microorganisms, and also reducing any adverse side effects [26].

The effects of seasons on the biochemical features of some EOs of the Lamiaceae family have been described in the literature [16, 27]. However, no information is available on how seasonal variations would affect the EO content, composition, and yields of the major oil constituents of spearmint cultivated in Palestine.

In Traditional Arabic Palestinian Herbal Medicine (TAPHM), the fresh and dried spearmint EOs have been used for the treatment of obesity, dementia, hypertension, abdominal pain, digestive disorders, muscle spasm, flatulence, headache, fever, menstrual pain, asthma, cough, cold, depression, and others [7, 16, 28–31]. In addition, they are commonly used as a memory enhancer and nerve sedative [32]. In animals, spearmint is used as a laxative, diuretic, hypothermia blocker, and flea repellent and for sore throat [33].

The objectives of this study were to (1) study the effect of seasons on the EO content and composition of spearmint cultivated in Palestine; (2) investigate the biological activities of the EOs focusing on their potential benefits in the treatment of obesity, Alzheimer's disease (AD), dermatophytosis, and antibiotic-resistant infections; and (3) verify the use of *M. spicata* EO as a folk medicine in TAPHM and open perspectives to find more effective substances from plant origin for the treatment of important human diseases.

## 2. Materials and Methods

### 2.1. Plant Material *M. spicata*

Samples of aerial parts were collected from a spearmint field in the BERC Experimental Station, Til, Nablus, Palestine, during three consecutive years (2015–2017). EOs were separated from the fresh aerial parts by hydrodistillation using a modified Clevenger apparatus following the conditions reported in [16].

### 2.2. Chemical Analysis of the Essential Oils

Determination of EO composition was performed using gas chromatography-mass spectrometry (GC-MS) following the conditions reported in [16, 27]. The identification of the constituents was based on comparison of their relative retention indices and spectra with spectra of NIST 98, QuadLib1607 GC-MS, and Adams libraries [34].

### 2.3. Determination of Antidermatophytic Activity

Essential oil and its main component, carvone, were tested for their antidermatophytic activity against four dermatophyte species: *Microsporum canis*, *Trichophyton mentagrophytes*, *T. rubrum*, and *Epidermophyton floccosum*, using the poisoned food technique as described by Gakuubi et al. [35] and Mohareb et al. [36]. EO and carvone were tested at concentrations ranging from 0.3 to 4 ml/L. Mycelial growth inhibition % (PI) was calculated as follows:

(1) $$\begin{array}{c} \text{mycelial growth inhibition \%}\left(\text{PI}\right)=\left[\frac{\text{DC}-\text{DT}}{\text{DC}}\right]\;\times 100, \end{array}$$

where DC and DT are average diameters of fungal growth in control and treatment groups, respectively. Effective concentration that caused 50% mycelial growth inhibition (EC_50_) was assessed using Excel.

Minimum inhibitory concentration (MIC) and minimum fungicidal concentration (MFC) were determined following previously reported assays [35, 37].

### 2.4. Determination of Antibiofilm Activity

#### 2.4.1. Activity against Biofilm Formation

A biofilm-producing bacterial strain, *MRSA*-*BERC #01*, was used to assess the potential of *M. spicata* EO and carvone to prevent biofilm formation. Aliquots of twofold serial dilutions (100 *μ*L) of EO and carvone were prepared in tryptic soy peptone supplemented with the 0.2% glucose (TSBGlc) medium and added to the 96-well flat-bottom microtiter plate, with final concentrations ranging from 0.078 to 5 *μ*L/mL. Bacterial suspensions (100 *μ*L; 1 × 10^6^ CFU/mL, final concentration) were then added to the plate. The TSBGlc medium was employed as a negative control. TSBGlc without essential oil was used as a blank [38, 39]. After incubation at 37°C for 24 h, the ability of *MRSA-BERC #01* to form the biofilm in the presence of carvone or EO was then determined using the 2,3,5-triphenyl-tetrazolium chloride (TTC) reduction and crystal violet (CV) assays as mentioned below.

#### 2.4.2. Effects on Established Biofilms

The effect of *M. spicata* EO and carvone on established biofilms was tested following Sabaeifard et al. [40]. In brief, 100 *μ*L of the bacterial suspension (1 × 10^6^ CFU/mL) was added to each well in the microtiter plate and incubated for 24 h at 37°C to allow biofilm formation. After incubation, 100 *μ*L of different concentrations of the EO was added to each well to give final concentrations ranging between 0.078 and 5 *μ*L/mL. TSBGlc was used as the negative control and the blank. The plates were incubated for 24 h after incubation, the planktonic cells were removed by gently aspirating the cell suspension, and the plate was washed twice with PBS. Extra moisture was removed by tapping the plate on a sterile tissue paper. The microplates were left to dry in a laminar flow desk for 15 min in the upside-down position. The activity of EOs or carvone on the preformed biofilm was subsequently determined using the TTC reduction assay and CV assay [41–45] as mentioned below.

#### 2.4.3. TTC Assay

Biofilm metabolic activity was measured using the TTC assay following Sabaeifard et al. [40]. In brief, fifty microliters of TTC (0.2%) mixed with 200 *μ*L of TSBGlc were transferred to each well, and the plates were then kept in the dark at 37°C for 4 h. Following incubation, the TTC solution was aspirated, and the plate was air-dried. Attached red formazan was dissolved in a mixture of acetone (20%) and ethanol (80%). The dissolved red formazan was moved to a new flat-bottom microplate, and absorbance was measured at 500 nm using Epoch Microplate Spectrophotometer (BioTek) [41–45].

#### 2.4.4. Crystal Violet Assay (CV Assay)

The effect of EOs on the total biofilm biomass was measured by the CV staining protocol as described by Peeters et al. [45]. In brief, the formed biofilm was washed using PBS, and the remaining biofilm was stained using 0.01% CV. The bound CV was dissolved with 33% acetic acid, and absorbance at 550 nm was measured using Epoch Microplate Spectrophotometer (BioTek).

### 2.5. Enzyme Inhibitory Activity

#### 2.5.1. Anticholinesterase Assays

Anticholinesterase activities against acetylcholinesterase (AChE) and butyrylcholinesterase (BuChE) were measured spectrophotometrically using the NA-FB method following Ali-Shtayeh et al. [4]. Neostigmine bromide and galanthamine hydrobromide were used as reference standards.

#### 2.5.2. Pancreatic Lipase Inhibition

The ability of the *M. spicata* EO to inhibit PPL was determined based on a spectrophotometric analysis as described previously [16]. Orlistat was used as the reference standard.

The interactions of EO or carvone with orlistat were determined using a checkerboard method as described by Nikkhah et al. [46]. A two-dimensional checkerboard with twofold dilutions of two treatment agents comprising *M. spicata* EO/orlistat and carvone/orlistat was carried out. The evaluated concentrations were in the range of 7 dilutions with final concentrations of 50, 25, 12.5, 6.25, 3.125, 1.56, and 0.78 *μ*L/mL for EO and carvone and 0.063, 0.031, 0.016, 0.008, 0.004, 0.002, and 0.001 *μ*g/mL for orlistat.

The fractional inhibitory concentration (FIC) was calculated for the first well in each row of the microtiter plate containing the combination with lipase percentage of inhibition more than 50%, given as follows: FIC of compound A (FIC_A_) = MIC_50_ (A) in combination/MIC_50_ (A) alone and FIC of compound B (FIC_B_) = MIC_50_ (B) in combination/IC_50_ (B) alone, where A is EO or carvone and B is orlistat.

The FIC index (FICI) was calculated as follows:

(2) $$\begin{array}{c} \text{FICI}=\left({\text{FIC}}_{\text{A}}+{\text{FIC}}_{\text{B}}\right). \end{array}$$

The obtained results were inferred as follows: synergistic effect (FICI ≤ 0.5), additive effect (0.5 < FICI ≤ 1), no interactive effect (1 < FICI ≤ 4), and antagonistic effect (FICI > 4) [47, 48]. The results were graphically represented as isobolograms using Excel. The isobolograms were used to define the type of interaction between EO or carvone and orlistat combination. The isobolograms were performed by mixing EO or carvone with orlistat to determine what lipase inhibitory interaction could be observed by different concentrations of EO and orlistat combined. The isobologram curves can be built by plotting the data points of different ratios where each MIC_50_ is defined in relation to the independent MIC_50_. A concave curve represents synergy, whereas convex curve and straight line indicate antagonistic and additive effects, respectively [49].

### 2.6. Statistical Analysis

Averages and standard deviations for three simultaneous assays were tested using standard statistical methods. The relationships between the studied phytochemical compositions were expressed by Pearson's correlation coefficient (SPSS software 21).

## 3. Results and Discussion

### 3.1. Seasonal Variations in *M. spicata* Essential Oil Content

The EOs obtained from *M. spicata* varied quantitatively according to seasons. *M. spicata* demonstrated a significantly higher (*p* ≤ 0.05) EO content in summer and fall months when the plants were in full bloom (2.79% in summer and 2.54% in fall) than that in winter and spring months when the plants have reached the end of their growing cycle (0.87–1.94%) (Table 1). The results demonstrated that EO accumulation in *M. spicata* seems to be metabolically controlled during vegetative and flowering stages of plant growth. Our results are in agreement with those of Hussain et al. [11] who also reported higher EO contents from late summer crops. A similar outcome was also obtained for *Origanum syriacum* and *Clinopodium serpyllifolium* which also showed the maximum EO yield during summer, when the plants were in full bloom [16, 27].

### 3.2. *Mentha spicata* EO Chemical Composition

**Figure 1:**
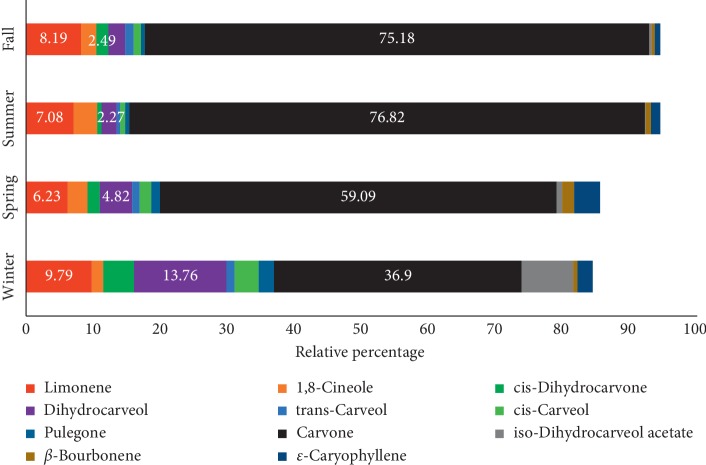
Seasonal variation of percentage of *M. spicata* EO major compounds over a 3-year study period.

The phytochemical analysis results of the EOs are detailed in Table 1. Thirty-one compounds were identified in the *M. spicata* EOs. Eleven of the components (limonene, 1,8-cineole, cis-dihydrocarvone, dihydrocarveol, trans-carveol, cis-carveol, pulegone, carvone, iso-dihydrocarveol acetate, *β*-bourbonene, and *ε*-caryophyllene) show mean percentages more than 1%, which represent 90% of the total oils (Figure 1). Three of these compounds were the major constituents: carvone (36.9–76.8%), limonene (6.23–9.79%), and dihydrocarveol (2.27–13.76%). Hence, regardless of the season, all *M. spicata* EOs (collected in all seasons) can be considered carvone chemotypes. Our results are in consistent with those of other scientists who also found the EO of *M. spicata* to be of the carvone chemotype [3, 12, 21, 50, 51].

The percentage of carvone in the EOs of *M. spicata* plants (Table 1 and Figure 1) increased gradually from winter to spring where it reached 59.09%; the percentage reached its maximum level of 76.82 and 75.18% in summer and fall months, respectively. The seasonal variations in phytochemical profiles of the EOs might be due to the impact of the phenological status and environmental conditions which can affect the regulation of the biosynthesis of EOs [11, 16, 52].

The Pearson correlation exhibited significant negative correlation between carvone and other phytochemical components (not presented). Carvone was negatively correlated with cis-carveol (*r* = −0.88), pulegone (*r* = −0.83), and dihydrocarveol (*r* = −0.79), indicating very high reverse correlation between carvone and these components. Lower percentages of carvone in EOs were shown during winter and spring months accompanied by an increased concentration of limonene, dihydrocarveol, pulegone, and cis-carveol.

The data shown in Table 1 demonstrate that the oxygenated monoterpenes (OMs) contained the highest percentages of all the tested EOs in the range of 84.8–95.3% over the study period, followed by sesquiterpene hydrocarbons in a range of 2.2–10.9% over the study period. Our results are consistent with those of Hussain et al. [11] who also found OMs to be dominant (81.48% and 78.33%) in *M. spicata* oils collected during summer and winter months, respectively, followed by monoterpene hydrocarbons and sesquiterpene hydrocarbons.

**Figure 2:**
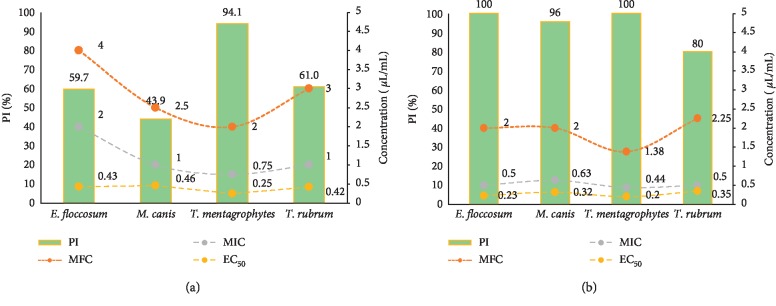
Percentage of mycelial growth inhibition (PI) of (a) *M. spicata* EO and (b) carvone against the test dermatophytes with MIC, MFC, and EC_50_ values.

Oxygenated monoterpenes generally followed a variation pattern (strong positive correlation between EO yield and OM percentage (*r* = 0.696)) comparable to that of EO yield with the highest OM percentages (94.7–95.3%) concurring with warmer seasons (summer and early fall) characterized by higher plant growth rates when the plants were in full bloom and with the lowest OM percentages (84.8–86.4%) coinciding with cooler weather conditions in winter and early spring characterized by lower plant growth rates when the plants reached the end of their growing season (Figure 2). These results are in agreement with those of Hussain et al. [11] who also described considerable seasonal variations in the content of the EO of *M. spicata*. This result would help scientists and industrialists to select ideal harvest time, allowing producing oil with high concentration of MOs. Since *M. spicata* EOs are rich in MOs, they are predicted to have high antifungal and enzyme inhibitory activities [53, 54].

### 3.3. *Mentha spicata* Antidermatophytic Activity

Dermatophytes are conceived to affect about 25% the world population [55–57]. The current treatment against dermatophytosis is based on synthetic antimycotic drugs. However, these artificial drugs are somewhat expensive in addition to having adverse reactions and slow action. The use of synthetic antifungal drugs, especially in case the drugs are not well managed, can increase the chances of recurrence and of selecting resistant strains [57, 58]. However, EOs obtained from aromatic plants are very vital natural products which have diverse therapeutic and biological activities, and EOs are known to be inhibitive or lethal to fungi and represent a potential source of new antifungal agents [53]. In view of the growing resistance to the conventional antimycotic agents, the EOs may be beneficial in the clinical management of mycosis, mainly dermatophytosis [59].

**Figure 3:**
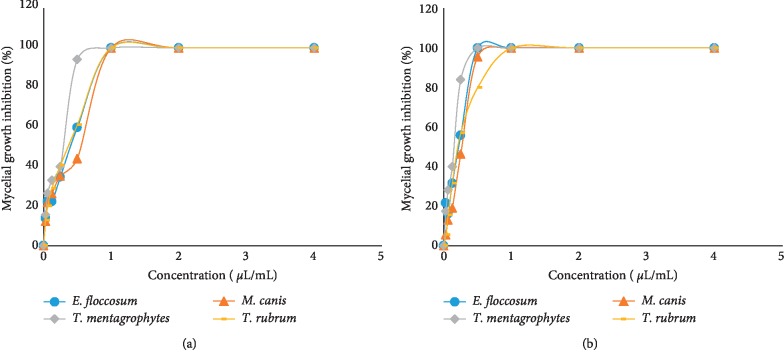
Mycelial growth inhibition activity of (a) *M. spicata* EO and (b) carvone against the test dermatophytes.

In the current study, *M. spicata* EO and carvone exhibited high antidermatophytic activity against *M. canis*, *T. rubrum*, *T. mentagrophytes*, and *E. floccosum* as indicated by PI, MIC, MFC, and EC_50_ (Figures 2 and 3).

The EO of *M. spicata* and its main compound (carvone) showed a dose-dependent activity against the test dermatophytes (Figure 3). Generally, as the dose of the EO or carvone increased, the antidermatophytic activity increased, represented by an increase in the mycelial growth inhibition. The results of PI values at different concentrations of oil and carvone are presented in Figure 2. The results indicated that the radial growth of all tested strains was entirely inhibited by the EO and carvone at 1, 2, and 4 *μ*L/mL concentrations. However, at lower doses (0.03–0.5 *μ*L/mL), the oil and carvone were generally more active on the mycelia growth of *T. mentagrophytes* than other tested dermatophytes; at 0.5 *μ*L/mL, PI = 94.1% and 100% for oil and carvone, respectively (Figure 2).

The MICs of the EO of *M. spicata* on the test dermatophytes ranged from 0.75 to 2 *μ*L/mL, and the EC_50_ of *M. spicata* EO ranged from 0.25 to 0.46 *μ*L/mL. However, EO showed fungicidal effect on the four studied dermatophytes, and the MFCs ranged from 2 to 4 *μ*L/mL. *T. mentagrophytes* was more susceptible to EO than the other tested fungi with MIC, MFC, and EC_50_ values of 0.44, 1.38, and 0.2 *μ*L/mL, respectively.

The strong antidermatophytic activity may be elucidated by the main component of the EOs, carvone, the oxygenated monoterpene which exhibited strong inhibitory activity against the tested dermatophytes (Figure 2) with PI, MIC, EC_50_, and MFC values ranging from 80 to 100%, 0.44 to 0.63 *μ*L/mL, 0.2 to 0.35 *μ*L/mL, and 1.38 to 2.25 *μ*L/mL, respectively. The monoterpene alcohols are known to have strong antidermatophytic activity because of their good solubility in water and the presence of the functional alcohol group. Monoterpene alcohols can exert their antidermatophytic effects by increasing the permeability of the plasma membrane, causing the disruption of the cell membrane, and inhibiting the process of respiration on the mitochondrial membrane of fungi leading to death of cells or inhibiting the spore production and germination of dermatophytic fungi [53, 60–63].

### 3.4. *Mentha spicata* Antibiofilm Activity

MRSA is a common cause of dermal and soft-tissue infections. MRSA has generated increasing concern because of restricted treatment alternatives since its strains are resistant to the whole class of *β*-lactam antibiotics [64]. Eradication of MRSA is not always successful because of its ability to form biofilms.

Biofilms are communities of sessile microorganisms that are embedded in an extracellular matrix of proteins, lipids, polysaccharides, and nucleic acids, which confers protection to bacteria against host defenses and inhibits the delivery of antimicrobial agents [65]. Biofilm-associated bacteria are much more resistant to antimicrobial agents. The discovery of anti-infective agents with antibiofilm activity represents an important objective. In fact, inhibition of the biofilm formation effect of *M. spicata* essential oil has been reported in the case of *Vibrio* spp., *Staphylococcus aureus*, and dental biofilm [12, 66, 67].

In the current study, we evaluated the ability of several doses of *M. spicata* EO and its main component (carvone) to inhibit or destroy biofilm on a polystyrene surface formed by MRSA.

#### 3.4.1. Effects on Biofilm Formation (Inhibition of Cell Attachment)

**Figure 4:**
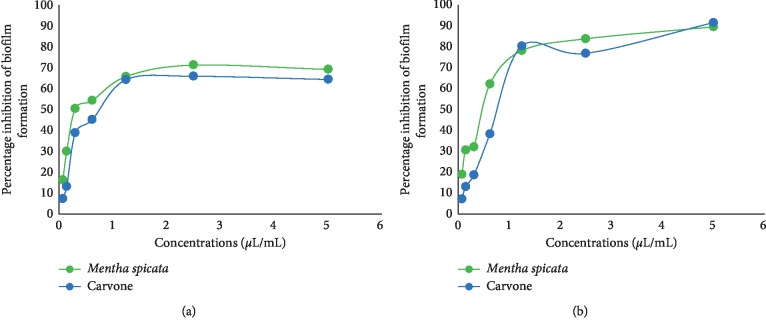
Effects of various doses *M. spicata* EO and carvone on biofilm formation indicated as % of inhibition, evaluated by the (a) crystal violet (CV) assay and (b) TTC assay.

Prevention of biofilm formation was examined on MRSA and assessed by crystal violet and TTC assays. The results were expressed as inhibition % of biofilm development. *M. spicata* EO exhibited a significant inhibitory activity on biomass and metabolic activity of MRSA biofilm formation in a dose-dependent manner (Figure 4). The crystal violet assay showed that *M. spicata* EO reduced the number of adherent bacteria (biomass) by 71.3% at 5 *μ*L/mL, and the observed biofilm inhibitory concentration (BIC_50_) was 0.37 *μ*L/mL (Table 2).

**Figure 5:**
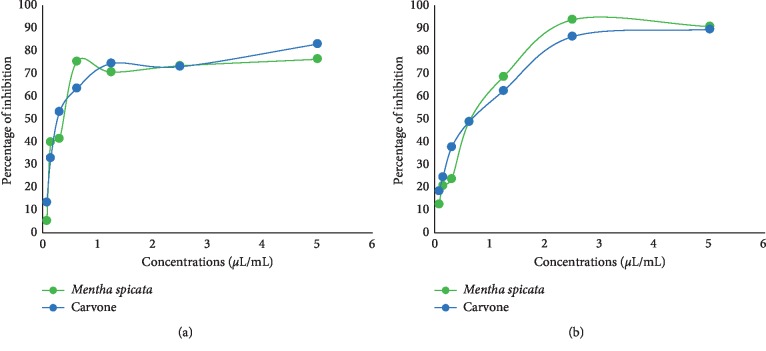
Effects of various doses of *M. spicata* EO and carvone on the preformed biofilm presented as % of inhibition, evaluated by the (a) crystal violet (CV) assay and (b) TTC assay.

Inhibition of biofilm formation by *M. spicata* EO was confirmed by the TTC assay; in the presence of *M. spicata* EO, the metabolic oxidative activity was noticeably decreased after 24 h of incubation by 89.5% at 5 *μ*L/mL concentration compared to the nontreated biofilm (Figure 5), and the observed BIC_50_ value was 0.41 *μ*L/mL (Table 2).

These results demonstrated that, in addition to the decreasing number of attached bacteria (biomass) evaluated by the CV assay, *M. spicata* EO has a strong inhibitory effect on metabolic activity of cells embedded in the biofilm.

#### 3.4.2. Effects on Preformed Biofilm

Essential oil from *M. spicata* and carvone showed significant effects on the biomass and oxidative metabolic activity of the preformed biofilm in a dose-dependent manner (Figure 5). *M. spicata* essential oil eradicated more than 75% of preformed biofilm biomass and metabolic activity at 5 *μ*L/mL. The BIC_50_ value observed by crystal violet and TTC assays was 0.39 and 0.89 *μ*L/mL, respectively.

The results demonstrate that, in addition to its activity against biofilm formation, *M. spicata* EO had a strong inhibitory effect on metabolic activity and biomass of the preformed biofilm. The significant activity of *M. spicata* EO on the MRSA biofilm may be elucidated by the major component of the EOs, carvone (Figures 4 and 5). Carvone has a strong effect on biofilm biomass and metabolic activity with the BIC_50_ value ranging between 0.34 and 0.68 *μ*L/mL (Table 2).

The success of *M. spicata* EO in inhibiting bacterial adhesion is a promising approach for treating infections of mucosal surfaces by reducing microbial colonization on surfaces and epithelial mucosa which leads to preventing the establishment of bacterial pathogenesis [68, 69].

### 3.5. Cholinesterase Inhibitory Activity of *M. spicata* Essential Oil

Several plant species from the Lamiaceae family are used in TAPHM to enhance memory [4, 31]. The ability of these plants to boost the cholinergic function by inhibiting cholinesterase is the most acceptable theory for their memory-enhancing activities [4]. In this study, the EO of *M. spicata* showed high levels of inhibitory activity against AChE and BuChE; the EO inhibited cholinesterase enzymes in a concentration-dependent manner, with AChE (IC_50_ = 23.1 *μ*L/mL) and BuChE (IC_50_ = 35.0 *μ*L/mL) inhibitory activities (Table 3), matching inhibitory activities of carvone (EO's main component) against AChE (IC_50_ = 19.01 *μ*L/mL) and BuChE (IC_50_ = 32.33 *μ*L/mL). It is therefore speculated that the anticholinesterase activity of the major monoterpenoid component (carvone) is accountable for the observed inhibitory effect, of the *M. spicata* aromatic plant [70, 71].

According to the results of the current study, it is highly recommended that the EO extracted from the aerial parts of *M. spicata* be further explored for possible useful effects on neurodegenerative disorders, such as AD, utilizing its cholinesterase inhibition activities.

### 3.6. Pancreatic Lipase Inhibitory Activity of *M. spicata* Essential Oil

**Figure 6:**
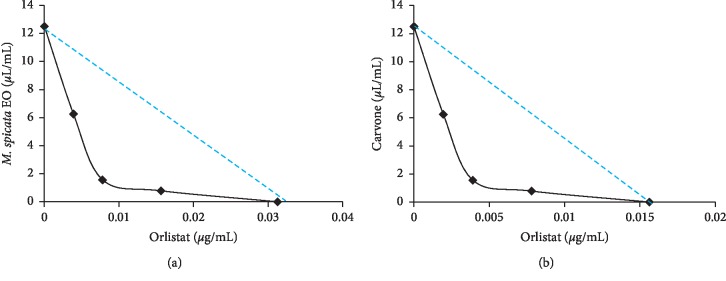
Isobologram curve of double combinations of EO or carvone with orlistat against porcine pancreatic lipase.

In the current study, *M. spicata* EO and its main component (carvone) were assessed for lipid-lowering activity through inhibition of porcine pancreatic lipase (PPL) (Table 4). *M. spicata* EO and carvone had a lipase inhibitory concentration (MIC_50_) of 12.5 *μ*L/mL. The fractional inhibitory concentrations (FICs) of the dual combinations of EO or carvone with orlistat are presented in Table 4. Both combinations displayed a synergistic effect against the lipase enzyme. The synergistic effect was presented graphically by using the isobologram method (Figure 6).

In this regard, the PPL inhibitory activities may in part be elucidated by the high content of carvone and other monoterpenes. The results support the view that *M. spicata* signifies a rich source of antilipase constituents. Jamous et al. [7] have previously reported that the ethanolic extracts of *M. spicata* exhibit a strong PPL inhibitory property with an IC_50_ of 1.19 mg/ml. However, to the best of our knowledge, EO from this plant or its phytochemicals have not been previously studied for their lipase inhibitory activity.

## 4. Conclusions

Because of its high biological activities, EO from *Mentha spicata* may be used for the development of new formulations of nutraceutical products for the management of chronic diseases such as Alzheimer's disease and overweight. *M. spicata* EO can fulfill the growing demand of industries for constant and good raw natural sources of antidermatophytic, anticholinesterase, and antiobesity agents that could be safer than synthetic drugs. To attain the optimum oil yield with a high quantity and content of OMs (particularly carvone), *M. spicata* should be extracted when the plants are in full bloom. The strong inhibitory activity of *M. spicata* EOs on AChE and PPL and antidermatophytic properties may be due to their high concentrations of carvone monoterpenes. The results also showed that, in addition to their significant inhibitory activity against biofilm formation of MRSA, *M. spicata* EO and carvone had a strong inhibitory effect on metabolic activity and biomass of the preformed biofilm. Besides the biological activities described here, the results support the use of *M. spicata* EO as a folk medicine. The results also provide further evidence for the utilization of *M. spicata* EO and its principal constituents as an important source of anticholinesterase, antiobesity, antidermatophytic, and anti-MRSA agents.

## Figures and Tables

**Table 1:** Constituents (%) of the EOs from cultivated *M. spicata* sampled over a three-year period.

RT	RI	Compound	Winter	Spring	Summer	Fall
5.418	939	*α*-Pinene	0.54 ± 0.05	0.35 ± 0.07	0.25 ± 0.03	0.32 ± 0.01
5.816	946	Camphene	0.16 ± 0	0.14 ± 0.02	0.07 ± 0.01	0.06 ± 0
6.393	969	Sabinene	5.51 ± 1.79	0.69 ± 0.25	0.14 ± 0.01	0.22 ± 0
6.518	974	*β*-Pinene	0.73 ± 0.06	0.59 ± 0.1	0.35 ± 0.01	0.41 ± 0
6.846	988	Myrcene	0.51 ± 0.06	0.36 ± 0.07	0.17 ± 0.01	0.23 ± 0
7.048	988	3-Octanol	0.46 ± 0.02	0.47 ± 0.02	0.35 ± 0.06	0.44 ± 0.03
7.809	1020	para-Cymene	0.27 ± 0.04	0.31 ± 0.01	0.33 ± 0.01	0.11 ± 0.02
7.927	1029	**Limonene**	**9.79** **±** **0.55**	**6.23** **±** **0.1**	**7.08** **±** **0.65**	**8.19** **±** **1.13**
7.99	1031	1,8-Cineole	1.72 ± 0.35	2.92 ± 0.18	3.52 ± 0.01	2.3 ± 0.14
8.8	1060	*γ*-Terpinene	0.25 ± 0.02	0.42 ± 0.09	0.05 ± 0	0.03 ± 0
9.086	1070	cis-Sabinene hydrate	1.11 ± 0.2	1.34 ± 0.37	0.69 ± 0.23	0.28 ± 0
12.072	1165	Borneol	0.56 ± 0.02	1.16 ± 0.03	0.66 ± 0.04	0.63 ± 0.05
12.341	1174	Terpinen-4-ol	1 ± 0.2	1.13 ± 0.31	0.47 ± 0.07	0.18 ± 0.01
12.53	1186	*α*-Terpineol	0.12 ± 0.03	0.19 ± 0	0.31 ± 0.01	0.25 ± 0
12.83	1191	cis-Dihydrocarvone	4.59 ± 0.39	1.84 ± 0.01	0.65 ± 0.18	1.79 ± 0.56
12.959	1192	Dihydrocarveol	13.76 ± 0.11	4.82 ± 0.2	2.27 ± 0.35	2.49 ± 1.4
13.511	1215	trans-Carveol	1.23 ± 0.33	1.13 ± 0.09	0.52 ± 0.02	1.28 ± 0.07
13.904	1226	cis-Carveol	3.57 ± 0.02	1.71 ± 0.02	0.73 ± 0.09	1.07 ± 0.07
14.12	1233	Pulegone	2.3 ± 0.16	1.32 ± 0.08	0.63 ± 0.06	0.58 ± 0.05
14.239	1239	**Carvone**	**36.9** **±** **2.67**	**59.09** **±** **0.74**	**76.82** **±** **0.87**	**75.18** **±** **2.54**
15.32	1287	Bornyl acetate	0.99 ± 0.07	0.3 ± 0.03	0.1 ± 0.01	0.39 ± 0.1
16.464	1329	iso-Dihydrocarveol acetate	7.67 ± 0.76	0.83 ± 0	0.12 ± 0.02	0.36 ± 0.07
16.692	1339	trans-Carvyl acetate	0.87 ± 0.02	0.35 ± 0.12	0.06 ± 0.04	0.31 ± 0.14
18.123	1387	*β*-Bourbonene	0.64 ± 0.12	1.78 ± 0.08	0.79 ± 0.02	0.46 ± 0.03
18.278	1389	*β*-Elemene	0.96 ± 0.06	1.48 ± 0.11	0.38 ± 0.04	0.3 ± 0
19.079	1417	*ε*-Caryophyllene	2.27 ± 0.16	3.87 ± 0.06	1.38 ± 0.15	0.81 ± 0.07
20.2	1430	*β*-Copaene	0.51 ± 0.18	0.77 ± 0.06	0.04 ± 0	0.23 ± 0.02
20.7	1484	Germacrene D	0.43 ± 0.17	1.57 ± 0.05	0.14 ± 0	0.15 ± 0
21.1	1500	Bicyclogermacrene	0.15 ± 0.09	1.03 ± 0.04	0.06 ± 0	0.09 ± 0.02
21.7	1529	trans-Calamenene	0.27 ± 0.13	0.39 ± 0.07	0.01 ± 0	0.11 ± 0.02
23.2	1582	Caryophyllene oxide	0.18 ± 0.03	1.45 ± 0.48	0.84 ± 0.09	0.73 ± 0.16

Essential oil yield (%)			0.87 ± 0.02	1.94 ± 0.04	2.79 ± 0.08	2.54 ± 0.09

Chemical group						
Monoterpene hydrocarbons			8.2 ± 2.69	2.9 ± 0.73	1.7 ± 0.18	1.8 ± 0.01
**Oxygenated monoterpenes**			**86.4** **±** **4.02**	**84.8** **±** **0.01**	**94.7** **±** **0.53**	**95.3** **±** **0.4**
Sesquiterpene hydrocarbons			5.2 ± 1.29	10.9 ± 0.04	2.8 ± 0.23	2.2 ± 0.17
Oxygenated sesquiterpenes			0.2 ± 0.05	1.4 ± 0.68	0.8 ± 0.12	0.7 ± 0.22

RT: retention time; RI: retention index.

**Table 2:** Antibiofilm effect of *Mentha spicata* EO and carvone against the MRSA-positive biofilm strain.

	Effect upon biofilm formation		Effect upon preformed biofilm	
	BIC_50_ (*μ*L/mL)			
	CV assay	TTC assay	CV assay	TTC assay
*Mentha spicata*	0.37 ± 0.05	0.41 ± 0.13	0.39 ± 0.11	0.89 ± 0.05
Carvone	0.53 ± 0.14	0.68 ± 0.1	0.34 ± 0.06	0.66 ± 0.02

**Table 3:** Cholinesterase inhibitory activities of essential oil from *M. spicata* growing in Palestine.

	IC_50_ (*µ*L/mL)	
	Acetylcholinesterase (AChE)	Butyrylcholinesterase (BuChE)
Essential oil	23.1 ± 0.26	35.0 ± 0.37
Carvone	19.01 ± 0.45	32.33 ± 0.09
Galanthamine	0.0076	0.229
Neostagmine	0.0008	0.104

**Table 4:** FIC and interaction effects of double combinations of EO or carvone with orlistat on pancreatic lipase inhibitory activities.

Combination (A/B)	MIC_50_ A (alone) (*μ*L/mL)	MIC_50_ B (alone) (*μ*g/mL)	MIC_50_ A (in the presence of B) (*μ*L/mL)	MIC_50_ B (in the presence of A) (μg/mL)	Checkerboard FIC index
EO/orlistat	12.5	0.032	0.078	0.0039	0.11
Carvone/orlistat	12.5	0.032	0.078	0.0019	0.065

## Data Availability

The datasets supporting the results of this study will be freely available upon request to the corresponding author (msshtayeh@yahoo.com) for noncommercial use only.

## Abbreviations

AChE: Acetylcholinesterase
AD: Alzheimer's disease
BERC: Biodiversity and Environmental Research Center
BuChE: Butyrylcholinesterase
EC_50_: Effective concentration with 50% inhibitory activity
TAPHM: Traditional Arabic Palestinian Herbal Medicine
IC_50_: Inhibitory concentration with 50% activity
MFC: Minimum fungicidal concentration
MIC: Minimum inhibitory concentration
MRSA: Methicillin-resistant *Staphylococcus aureus*
OMs: Oxygenated monoterpenes
PI: Percent of inhibition
PPL: Porcine pancreatic lipase
RI: Retention index
RT: Retention time.
